# An improved nucleic acid extraction method from dried blood spots for amplification of *Plasmodium falciparum kelch13* for detection of artemisinin resistance

**DOI:** 10.1186/s12936-019-2817-8

**Published:** 2019-06-11

**Authors:** Kayvan Zainabadi, Myaing M. Nyunt, Christopher V. Plowe

**Affiliations:** 1 Institute for Global Health, University of Maryland School of Medicine, Baltimore, MD USA; 2 Duke Global Health Institute, Duke University, Durham, NC USA; 3 Present Address: Center for Global Health, Weill Cornell Medicine, New York, NY USA

**Keywords:** Malaria, *Plasmodium falciparum*, Kelch13, K13, Drug resistance, Artemisinin resistance, Dried blood spot, PCR, Molecular surveillance, Asymptomatic infection, Diagnostics, Southeast Asia, DBS, Low transmission, Low parasitaemia

## Abstract

**Background:**

Mutational analysis of the *Plasmodium falciparum kelch 13* (*k13*) gene is routinely performed to track the emergence and spread of artemisinin resistance. Surveillance of resistance markers has been impeded by the difficulty of extracting sufficient DNA from low parasite density infections common in low-transmission settings, such as Southeast Asia. This problem can be overcome by collecting large volumes of venous blood. Efficient methods for extracting and amplifying *k13* from dried blood spots (DBS) would facilitate resistance surveillance.

**Methods:**

Methods for *k13* amplification from standard Whatman 3MM DBS (stored for 14 days at 28 °C with 80% relative humidity) were optimized by systematically testing different extraction conditions. Conditions that improved parasite DNA recovery as assessed by quantitative polymerase chain reaction (PCR) of 18S rDNA were then tested for their impact on *k13* PCR amplification.

**Results:**

The optimized protocol for amplification of *k13* from DBS is markedly more sensitive than standard methods using commercial kits. Using this method, *k13* was successfully amplified from laboratory-created DBS samples with parasite densities as low as 500 parasites/mL. Importantly, the method recovers both DNA and RNA, making it compatible with RNA-based ultrasensitive techniques currently in use.

**Conclusions:**

The optimized DBS protocol should facilitate drug resistance surveillance, especially in low-transmission settings where clinical malaria infections with high parasite densities are rare.

**Electronic supplementary material:**

The online version of this article (10.1186/s12936-019-2817-8) contains supplementary material, which is available to authorized users.

## Background

The emergence of multidrug resistance in Southeast Asia has led to high rates of treatment failure for first-line artemisinin-based combination therapy, raising the spectre of untreatable malaria in this region and threatening prospects for malaria elimination [1–7]. The discovery of molecular markers *kelch13* (*k13*) and *plasmepsin2/3* for resistance to artemisinin and piperaquine, respectively, has provided valuable tools for tracking resistance [8–10]. Artemisinin resistance is both spreading and arising independently in Southeast Asia, highlighting the pressing need for surveillance to guide treatment policies [11, 12].

The low parasitaemias associated with malaria infections in low-transmission settings has hindered surveillance efforts. Clinical infections from these regions are rare, and tend to present with much lower parasite densities than those found in high-transmission settings, such as Africa. Asymptomatic infections, while more common, have even lower parasite densities, necessitating the development of new ‘ultrasensitive’ techniques to identify them in these settings [13, 14]. These new techniques achieve vastly improved sensitivity (with lower limits of detection in the 10 s of parasites/mL) either by collecting high-volume blood samples (i.e., venous blood) or by amplifying highly abundant targets such as 18S rRNA [13, 14]. Importantly, these methods have revealed a large reservoir of previously unrecognized ‘silent’ malaria in Southeast Asia that vastly outnumbers clinically apparent cases [14–17].

Although more sensitive, these new methods also have drawbacks. For one, cumbersome sampling strategies, such as collection of venous blood and the need for a cold chain, limit their use in remote settings where most malaria is found. In addition, while the same eluate used for ultrasensitive detection can be used to characterize drug resistance markers, ultrasensitive methods that rely on amplification of RNA targets are not optimized for recovery of parasite DNA.

The ability to use dried blood spots (DBS) to detect drug resistance markers would facilitate surveillance efforts. A more sensitive DBS-based method would be particularly useful in low-transmission settings, both for passive surveillance (where lower parasitaemias trigger fever earlier in the course of infection in comparatively non-immune individuals) resulting in lower parasite densities at the time of case detection; and, in active surveillance (i.e., cross-sectional surveys), where parasitaemias tend to be even lower in asymptomatic carriers. This report describes a novel extraction method for DBS that markedly improves the success rate for PCR amplification of *k13*.

## Methods

### Mock dried blood spots

To create *Plasmodium falciparum* DBS, synchronous ring-stage cultured parasites of strain NF54 were obtained by a combination of sorbitol synchronization and magnetic separation. Parasites were quantified by microscopy by three independent microscopists and then diluted in parasite culture media followed by a final dilution in human whole blood. Samples were thoroughly mixed and then aliquoted (50 µL) onto Whatman 3MM filter paper. To simulate field conditions, samples were stored for 14 days at 28 °C with 80% relative humidity prior to extraction.

### Optimization of extraction conditions

A silica and chaotropic salt method was chosen for its simplicity, cost, and powerful ability to purify nucleic acids from complex mixtures while denaturing nucleic acid degrading enzymes. Nunc 96 Deep Well DNA filter plates (Sigma-Aldrich, St. Louis, MO, USA) were used for nucleic acid binding, though the less expensive Omega EZ DNA (VWR, Radnor, PA, USA) plates were found to give similar, albeit slightly reduced, sensitivity. Samples were incubated in a shaking incubator set to 60–65 °C and 250 RPM to assist in reconstituting the DBS with lysis buffer. Triton X-100 detergent was included (2% final volume/volume) to help release nucleic acids from the DBS and to keep denatured proteins in solution. EDTA (10 mM) was included to inhibit degradation of nucleic acids. A reducing environment was maintained by addition of 2-mercaptoethanol at a final concentration of .5% to further inactivate DNase and RNase enzymes and keep haemoglobin proteins in solution. Finally, the pH was buffered by addition of 5 mM Trizma HCl pH 7.4 and lowered to between 6 and 6.5 by addition of .1% 6 N HCl. Various different conditions were tested empirically by extracting laboratory-created DBS samples and analysing their effect by quantitative PCR for parasite 18S rDNA (described below). The final working protocol is presented as Additional file 1. Extraction using QIAamp or Investigator Kits (Qiagen, Valencia, CA, USA) was performed according to manufacturer’s instructions.

### PCR conditions

Quantitative PCR for *P. falciparum* 18S rDNA and human actin was performed as a multiplexed reaction using Qiagen QuantiTect multiplex RTPCR master mix and a Roche LC96 instrument. In the case of reverse-transcriptase PCR, Qiagen RT-MIX was added to the master mix and a RT step consisting of 50 °C for 20 min was included prior to PCR. All cycling conditions and primer/probe sequences were identical as previously reported [14]. Experiments were typically performed with at least three biological replicates and two technical replicates. An identical *k13* PCR protocol was followed as previously reported [8]. PCR products were run on 2% agarose gels and stained with ethidium bromide.

## Results

A systematic approach was taken to improve the extraction efficiency of DNA from DBS. To partially simulate field conditions, DBS samples were first stored at 28 °C with 80% relative humidity for 2 weeks prior to use. To assess the impact of different extraction conditions on DNA recovery, a multiplexed quantitative polymerase chain reaction (qPCR) strategy for detection of *P. falciparum* 18S rDNA and human actin was used. This initial screening strategy identified the following conditions that markedly improved parasite DNA recovery: (1) use of the stronger chaotropic salt guanidine thiocyanate (GuSCN) (vs guanidine hydrochloride) (Table 1); (2) inclusion of 16.7% (volume/volume) isopropanol in the lysis buffer **(**Tables 1, 2); (3) lowering the pH of the lysis buffer to below 6.5 (Table 3); and, (4) incubating the DBS at 65 °C for at least 1 h with lysis buffer (Table 4, Additional file 2).

**Table 1:** Guanidine thiocyanate (with alcohol) outperforms guanidine hydrochloride for extraction of DNA from dried blood spots

	3M GuSCN			3M GHCl		
Alcohol	0%	16.7% ISOH	16.7% ETOH	0%	16.7% ISOH	16.7% ETOH
Average Ct for Pf 18S rDNA (± SD)	Undetectable	29.2 (± .1)	29.3 (± .2)	Undetectable	Undetectable	Undetectable
Average Ct for human actin (± SD)	28.8 (± .2)	27.3 (± .3)	27.6 (± .3)	29.6 (± .4)	Undetectable	Undetectable

Experiments done with samples at parasite density of 2000 parasites/mL

Pf, *Plasmodium falciparum*; GuSCN, guanidine thiocyanate; GHCl, guanidine hydrochloride; ISOH, isopropanol; ETOH, ethanol; SD, standard deviation; Ct, cycle threshold

**Table 2:** Addition of 16.7% isopropanol to the lysis buffer improves recovery of parasite DNA

	3M GuSCN		
ISOH	10%	16.7%	25%
Average Ct for Pf 18S rDNA (± SD)	33.9 (± .4)	27.7 (± .2)	29.4 (± .2)
Average Ct for human actin (± SD)	27.0 (± .4)	26.5 (± .2)	26.8 (± .4)

Experiments done with samples at parasite density of 10,000 parasites/mL

Pf, *Plasmodium falciparum*; GuSCN, guanidine thiocyanate; ISOH, isopropanol; SD, standard deviation; Ct, cycle threshold

**Table 3:** A pH 6.5 or below for the lysis buffer leads to improved recovery of parasite DNA

pH	7.5	6.5	5.5
Average Ct for Pf 18S rDNA (± SD)	28.6 (± .6)	27.6 (± .1)	27.7 (± .3)
Average Ct for human actin (± SD)	26.2 (± .2)	26.2 (± .2)	26.6 (± .6)

Experiments done with samples at parasite density of 10,000 parasites/mL

Pf, *Plasmodium falciparum*; GuSCN, guanidine thiocyanate; ISOH, isopropanol; SD, standard deviation; Ct, cycle threshold

**Table 4:** Incubation at 65 °C for 1 h is superior to overnight incubation for recovery of parasite DNA from dried blood spots

Incubation time at 65 °C	1 h		Overnight	
Lysis buffer	3 M GuSCN	3 M GuSCN + 16.7% ISOH	3 M GuSCN	3 M GuSCN + 16.7% ISOH
Average Ct for Pf 18S rDNA (± SD)	Undetectable	30.0 (± .3)	Undetectable	Undetectable
Average Ct for human actin (± SD)	29.8 (± .4)	28.3 (± .3)	28.4 (± .3)	26.5 (± .9)

Experiments done with samples at parasite density of 2000 parasites/mL

Pf, *Plasmodium falciparum*; GuSCN, guanidine thiocyanate; ISOH, isopropanol; SD, standard deviation; Ct, cycle threshold

One unexpected finding from these experiments was that conditions that improved extraction efficiency (i.e., lowered the cycle threshold [*Ct*] values) for the *P. falciparum* target did not necessarily have the same effect on the human target. In most instances, human *actin Ct* values stayed relatively constant, and in some instances even showed an inverse relationship (Table 4). As a consequence, only *P. falciparum Ct* values were used for subsequent optimization experiments.

The lysis solution obtained after this initial optimization was similar to a buffer that the current authors previously reported for a DBS-based ultrasensitive method for detection of parasite 18S rRNA [17]. Side-by-side analysis showed that the two buffers performed equivalently for purification of both parasite DNA and RNA (Table 5). Further, the earlier study reported that commercially available RLT-plus buffer (with addition of 16.7% isopropanol v/v and .5% 2-mercaptoethanol) can be used as a substitute for the homemade lysis buffer. This buffer was tested and found to also perform equivalently (Additional file 3). Given that the current method purifies both RNA and DNA with high efficiency, it is suitable for use in RNA-based ultrasensitive methods, as well as characterization of parasite genomic targets.

**Table 5:** The final optimized lysis buffer performs similarly for purification of parasite DNA and RNA as a previously reported new extraction method (NEM) for RNA-based ultrasensitive detection of malaria from dried blood spots [17]

	3 M GuSCN + 16.7% ISOH			NEM lysis buffer		
Reverse-transcriptase	No	Yes	∆CT	No	Yes	∆CT
Average Ct for Pf 18S rDNA (± SD)	27.7 (± .2)	21.6 (± .3)	6.1	27.6 (± .1)	21.4 (± .2)	6.2

Experiments done with samples at parasite density of 10,000 parasites/mL

Pf, *Plasmodium falciparum*; GuSCN, guanidine thiocyanate; ISOH, isopropanol; SD, standard deviation; Ct, cycle threshold

Next, a comparison was made to determine how this protocol compares to a Qiagen QIAamp kit-based method. As is evident in Table 6, the current method resulted in a 5 *Ct* value improvement for the Pf 18S rDNA target in comparison to Qiagen QIAamp, which equates to a roughly 30-fold improvement in DNA recovery. This was evident for DBS samples stored on both Whatman 3MM and 903 Protein Saver, which performed equivalently.

**Table 6:** The current protocol outperforms Qiagen QIAamp for purification of parasite DNA from dried blood spots

Method	Current protocol		Qiagen QIAamp	
Filter paper	Whatman 3MM	Whatman 903 PS	Whatman 3MM	Whatman 903 PS
Average Ct for Pf 18S rDNA (± SD)	30.2 (± .1)	30.2 (± .2)	35.1 (± .1)	35.0 (± .1)

Experiments done with samples at parasite density of 2000 parasites/mL

Pf, *Plasmodium falciparum*; PS, protein saver; SD, standard deviation; Ct, cycle threshold

**Fig. 1:**
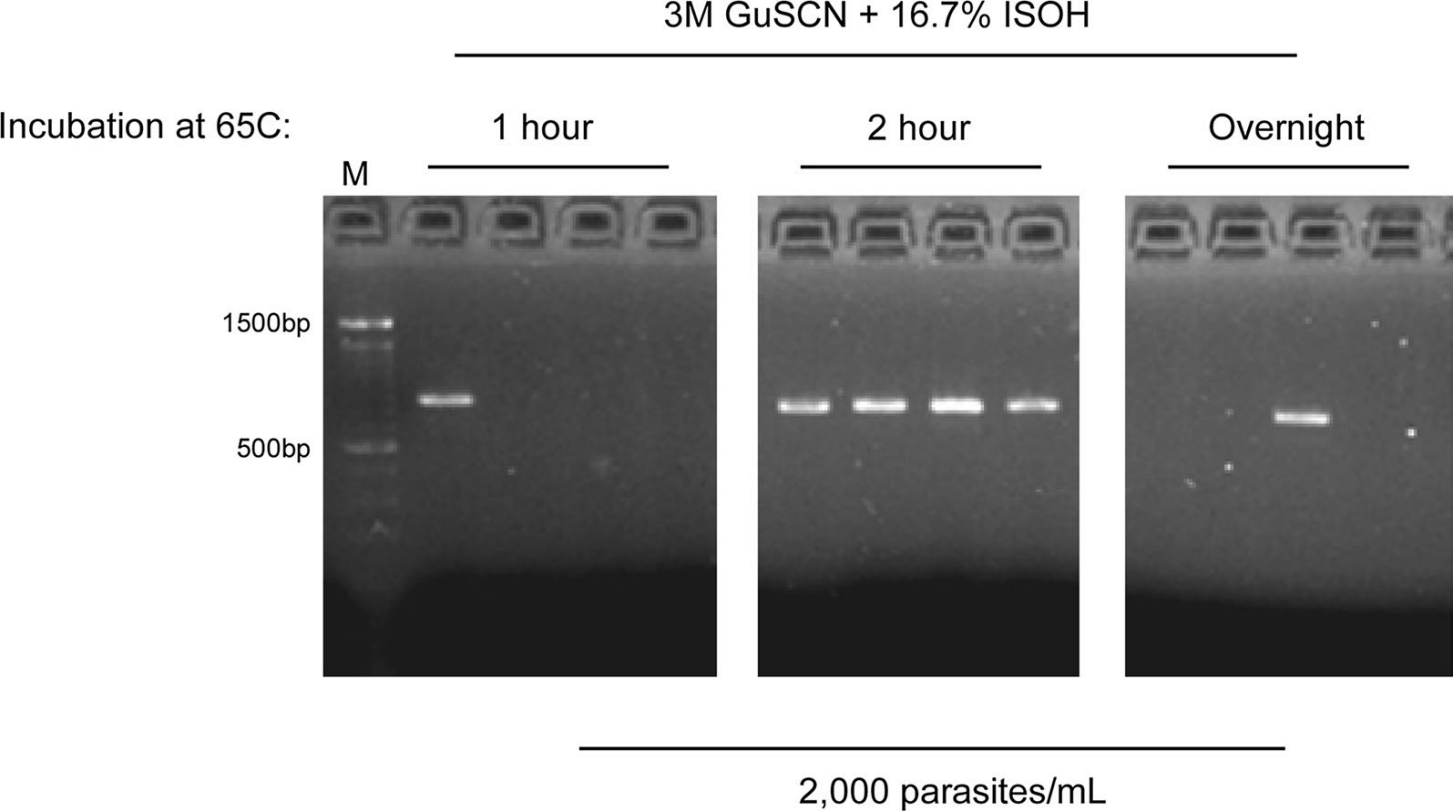
Incubating dried blood spots with lysis buffer at 65 °C for 2 h improves *k13* PCR success rate. *M* marker, *GuSCN* guanidine thiocyanate, *ISOH* isopropanol

**Fig. 2:**
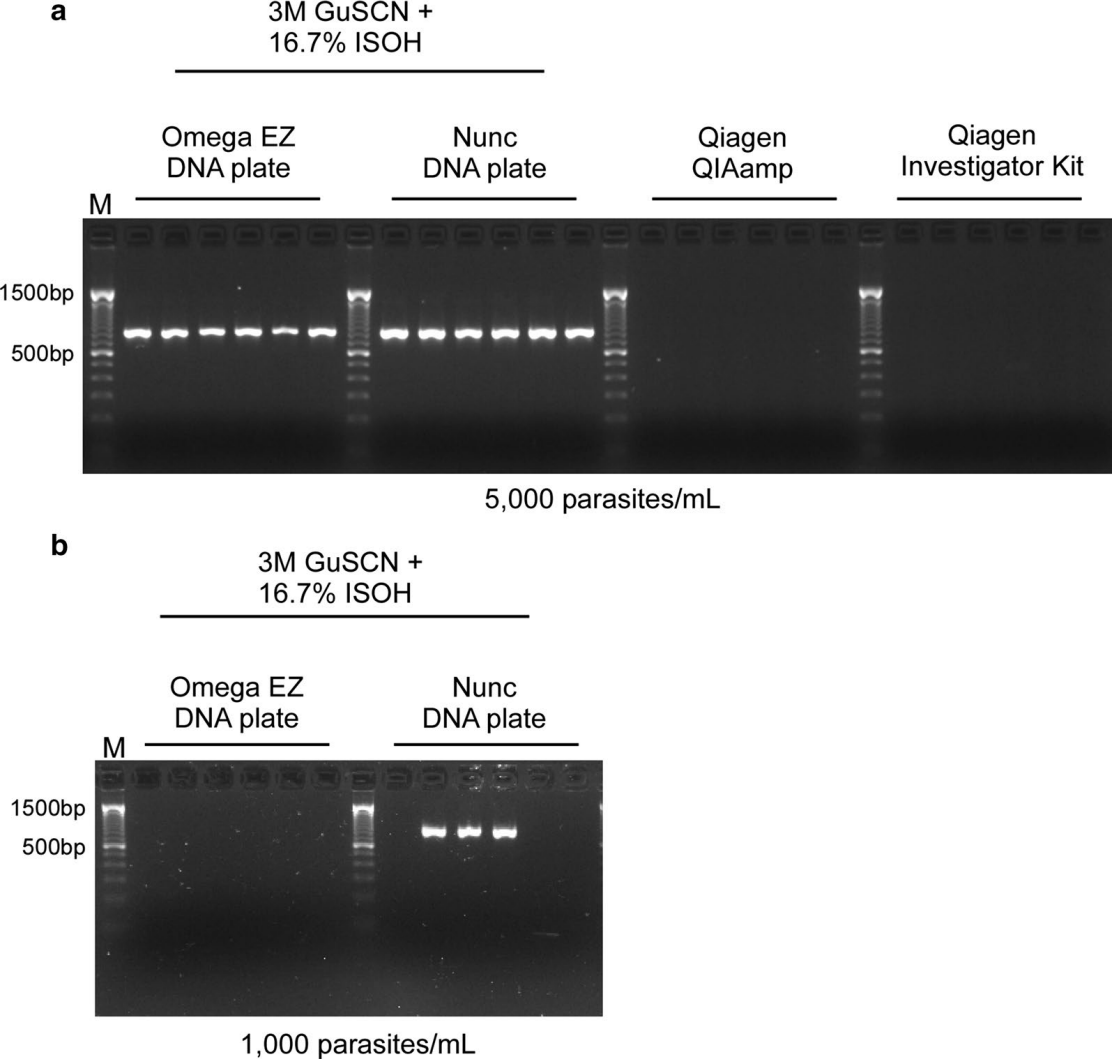
The current method outperforms Qiagen based kits for amplification of *k13* from dried blood spots. **a** The optimized protocol (compatible with Omega EZ and Nunc 96-well DNA plates) is able to amplify *k13* from all 5000 parasites/mL DBS samples, whereas Qiagen QIAamp and Investigator kits cannot. **b** Nunc DNA plates show improved sensitivity for amplification of 1000 parasites/mL DBS samples than Omega EZ DNA plates. *M* marker, *GuSCN* guanidine thiocyanate, *ISOH* isopropanol

This method was then tested using the *k13* PCR protocol. Given the difference in PCR strategy and amplicon size, conditions identified with the qPCR strategy were first validated for their performance in the *k13* assay. A key finding from this round of testing was that a longer incubation time of two hours (at 65 °C) markedly improved the success rate for *k13* amplification **(**Fig. 1**)**. This final protocol (Additional file 1) was then compared to the most widely used commercially available kits for DBS extraction. As can be seen in Fig. 2, the current protocol dramatically outperformed Qiagen QIAamp and Investigator kits, amplifying all 5000 parasites/mL DBS samples (whereas the kits failed completely).

**Fig. 3:**
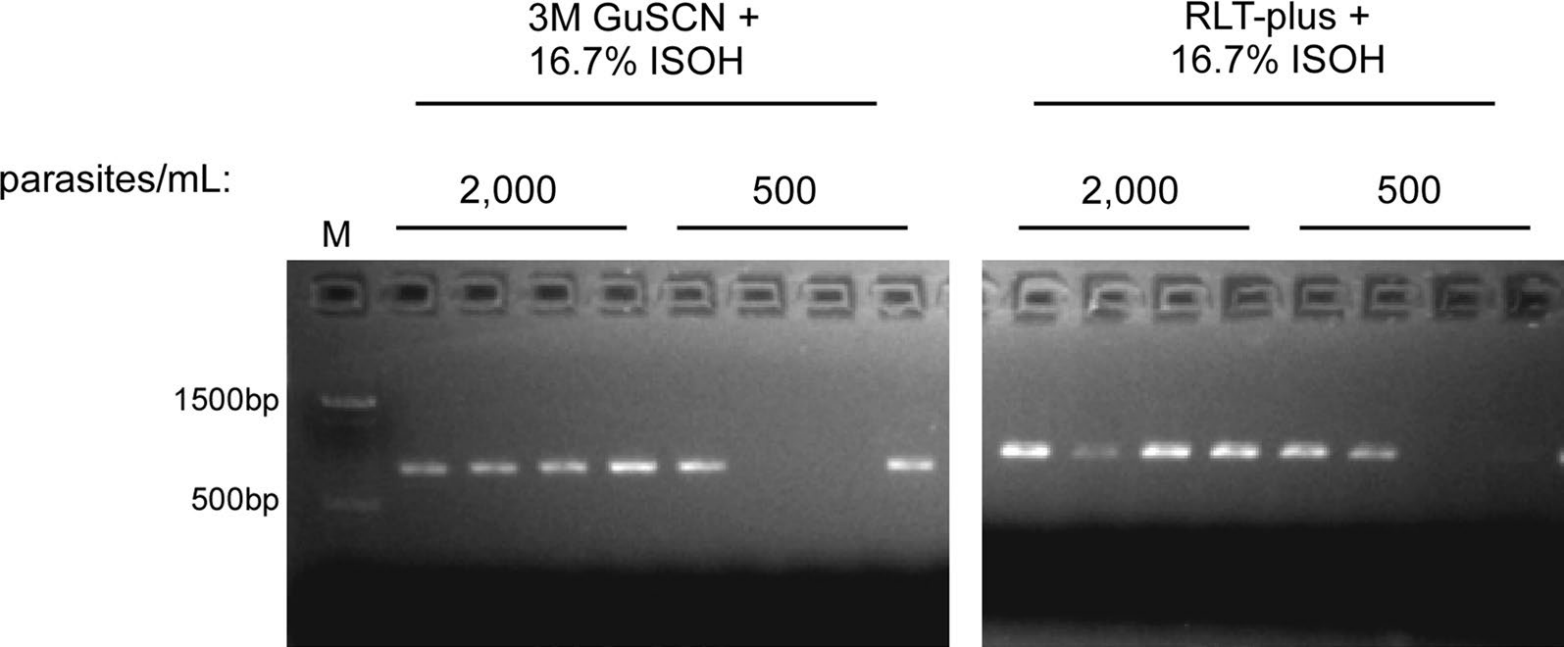
RLT-plus and home-made lysis buffers perform equally well for amplification of *k13* from dried blood spots. *M* marker, *GuSCN* guanidine thiocyanate, *ISOH* isopropanol

Finally, the RLT-plus buffer was also tested alongside the current protocol (that utilizes home-made buffers). The two methods performed identically, amplifying 4/4 of the 2000 parasites/mL samples and 2/4 of the 500 parasites/mL samples (Fig. 3). These data indicate that it may be possible to amplify DNA targets from samples containing as few as 10 parasites per 50 µL DBS sample.

## Discussion

In this report, a novel and more sensitive DBS-based method for detection of artemisinin resistance marker *kelch13* is presented. As the original *k13* PCR strategy was not altered [8], the improvement in sensitivity was achieved by optimizing the extraction efficiency from DBS. This was performed by taking a two-step approach: first, by using a qPCR strategy for an established parasite marker such as 18S rDNA, extraction conditions that incrementally improved parasite DNA recovery were identified; second, these conditions were combined and then tested for their impact on the *k13* PCR assay. In nearly all instances, conditions that improved DNA recovery in the first step also performed similarly in the second. However, in one instance, that of incubation time, it was found that a 2 h incubation at 65 °C was superior to 1 h. This appears to be specific for the *k13* PCR, as a 2-h incubation did not improve the quantitative recovery of 18S rDNA as assessed by qPCR (Additional file 2) or 18S rRNA as previously reported [17]. Importantly, overnight incubations were found to be highly detrimental in all instances and thus should be avoided.

This new protocol vastly outperforms Qiagen kit-based methods currently used for DBS, allowing for the successful amplification of parasitaemias as low as 500 parasites/mL. This may have ramifications for current surveillance efforts for drug resistance in Southeast Asia and other low-transmission settings where parasitaemias tend to be low in both clinical and asymptomatic infections, as compared to higher transmission settings [15, 16]. A recent study reported an average parasite density of approximately 5000 parasites/mL for asymptomatic *P. falciparum* infections in Southeast Asia [16]. It is, therefore, notable that the current protocol was able to amplify all DBS samples containing 5000 and 2000 parasites/mL, respectively.

For future studies, it will be important to determine how this method performs in samples obtained from the field. While this study attempted to control for stability by storing laboratory created samples in simulated field conditions for 2 weeks, the effect of longer exposure time on the *k13* assay is currently unknown. Running the extracted eluates obtained from DBS (and equivalent samples stored in preservative) shows that degradation clearly occurs for samples stored on filter paper (Additional file 4). Therefore, it would be prudent to store DBS samples at − 20 °C for long-term storage as this has previously been shown to prevent sample degradation [18]. However it should be noted that as long as degradation does not exceed the ~ 2 kb fragment size corresponding to the initial *k13* PCR amplicon, it should have minimal effect on amplification success. For instance, even though degradation is evident in the DBS eluates in Additional file 4, enough parasite DNA survived to successfully amplify *k13*. It is thus reasonable to anticipate that the current method can be applied to archival DBS material to better understand historic malaria trends, including those pertinent to drug resistance.

A surprising finding was that the final optimized conditions nearly mirrored a recently developed RNA-based ultrasensitive extraction method for DBS [17]. As utilizing one extraction protocol for purification of both RNA (for ultrasensitive detection) and DNA (for molecular characterization of DNA markers) would be ideal, the two methods were tested side-by-side and found to be perform identically. The same was true for the commercially available RLT-plus lysis buffer substitute, which may allow for easier adoption of the current method, particularly for use in multi-centred studies or in places where home-made solutions are not possible. It will be interesting to determine how this method fares for use in DNA-based ultrasensitive PCR techniques as well [13].

In addition to improved sensitivity, the described method is also high-throughput and cost-effective. The new method is 4.5 and 8.7 times less expensive than Qiagen QIAamp and Investigator kits, respectively (the latter of which is not high-throughput) (Table 7). Further, the collection and shipment of DBS is also markedly less expensive and cumbersome than whole blood-based methods. These qualities make the DBS method presented here ideal for use in large-scale surveillance by public health laboratories.

**Table 7:** The described method is markedly less expensive than commercially available kit-based methods

	Home-made buffers		RLT-plus buffers		Qiagen kits	
	Omega EZ DNA plate	Nunc DNA plate	Omega EZ DNA plate	Nunc DNA plate	QIAamp	Investigator
High-throughput?	Yes	Yes	Yes	Yes	Yes	No
Cost per sample	$1.77	$1.93	$2.66	$2.82	$7.87	$15.41

The increased sensitivity of the Nunc DNA plates comes at slightly increased cost. Based on list prices for all consumables for 10,000 extractions

It is important to note that as sensitivity increases, specificity often decreases. Previous reports have highlighted the use of strict contamination control measures to prevent cross-contamination of samples, particularly during cutting of the DBS samples [17]. Given the increased sensitivity of the current assay, it is recommended that such precautions be utilized.

Finally, the method described here may enable other applications from DBS. For example, it may prove useful for detection of other resistance markers, such as *plasmepsin2/3* for piperaquine resistance. Recent reports show that whole-genome sequencing is now possible from DBS, albeit only for high-density infections [19]. Therefore, it will be interesting to determine whether use of this optimized protocol will allow for sequencing from lower density infections as well. Improved purification of nucleic acids from DBS may have broader applications for other blood borne pathogens and disease states, helping to facilitate a shift towards DBS sampling for surveillance of diseases of global health importance.

## Conclusions

The use of dried blood spots should help facilitate scaling up current surveillance efforts to monitor the prevalence and distribution of drug-resistant malaria. As artemisinin-based combination treatment failure spreads within and potentially beyond Southeast Asia, robust surveillance methods are needed. Expanded surveillance will also facilitate evaluation of the contribution of drug-based elimination interventions, such as mass drug administration and screen-and-treat strategies, to the emergence and spread of resistance.

## Additional files

**Additional file 1.** The final* k13* dried blood spot (DBS) extraction protocol.

**Additional file 2.** Longer incubation times improve recovery of human, but not parasite DNA.

**Additional file 3.** Commercially available Qiagen RLT-plus buffer with 16.7% isopropanol performs similarly as the home-made lysis buffer.

**Additional file 4.** Evidence of nucleic acid degradation from samples stored on filter paper.

## Data Availability

Data sharing not applicable to this article as no datasets were generated or analyzed during the current study. A detailed Standard Operating Procedure is shared as Additional file 1, and the authors are willing to provide advice to researchers wishing to adopt the reported methods.

## Abbreviations

*Ct*: cycle threshold
DBS: dried blood spots
EDTA: ethylenediaminetetraacetic acid
HCl: hydrochloric acid
*K13*: *P. falciparum kelch 13* gene
mL: millilitre
PCR: polymerase chain reaction
RPM: revolutions per minute
RTPCR: reverse transcription PCR
rDNA: ribosomal DNA
rRNA: ribosomal RNA

## References

[1] Noedl H, Se Y, Schaecher K, Smith BL, Socheat D, Fukuda MM (2008). Artemisinin resistance in Cambodia 1 (ARC1) study consortium. Evidence of artemisinin-resistant malaria in western Cambodia. N Engl J Med..

[2] Amaratunga C, Sreng S, Suon S, Phelps ES, Stepniewska K, Lim P (2012). Artemisinin-resistant *Plasmodium falciparum* in Pursat province, western Cambodia: a parasite clearance rate study. Lancet Infect Dis..

[3] Dondorp AM, Nosten F, Yi P, Das D, Phyo AP, Tarning J (2009). Artemisinin resistance in *Plasmodium falciparum* malaria. N Engl J Med.

[4] Chaorattanakawee S, Saunders DL, Sea D, Chanarat N, Yingyuen K, Sundrakes S (2015). Ex vivo drug susceptibility testing and molecular profiling of clinical *Plasmodium falciparum* isolates from Cambodia from 2008 to 2013 suggest emerging piperaquine resistance. Antimicrob Agents Chemother.

[5] Leang R, Taylor WR, Bouth DM, Song L, Tarning J, Char MC (2015). Evidence of *Plasmodium falciparum* malaria multidrug resistance to artemisinin and piperaquine in western Cambodia: Dihydroartemisinin-piperaquine open-label multicenter clinical assessment. Antimicrob Agents Chemother.

[6] Spring MD, Lin JT, Manning JE, Vanachayangkul P, Somethy S, Bun R (2015). Dihydroartemisinin-piperaquine failure associated with a triple mutant including kelch13 C580Y in Cambodia: an observational cohort study. Lancet Infect Dis..

[7] Amaratunga C, Lim P, Suon S, Sreng S, Mao S, Sopha C (2016). Dihydroartemisinin-piperaquine resistance in *Plasmodium falciparum* malaria in Cambodia: a multisite prospective cohort study. Lancet Infect Dis..

[8] Ariey F, Witkowski B, Amaratunga C, Beghain J, Langlois AC, Khim N (2014). A molecular marker of artemisinin-resistant *Plasmodium falciparum* malaria. Nature.

[9] Witkowski B, Duru V, Khim N, Ross LS, Saintpierre B, Beghain J (2017). A surrogate marker of piperaquine-resistant *Plasmodium falciparum* malaria: a phenotype-genotype association study. Lancet Infect Dis..

[10] Amato R, Lim P, Miotto O, Amaratunga C, Dek D, Pearson RD (2017). Genetic markers associated with dihydroartemisinin-piperaquine failure in *Plasmodium falciparum* malaria in Cambodia: a genotype-phenotype association study. Lancet Infect Dis..

[11] Takala-Harrison S, Jacob CG, Arze C, Cummings MP, Silva JC, Dondorp AM (2015). Independent emergence of artemisinin resistance mutations among *Plasmodium falciparum* in Southeast Asia. J Infect Dis.

[12] Ménard D, Khim N, Beghain J, Adegnika AA, Shafiul-Alam M, Amodu O (2016). A worldwide map of *Plasmodium falciparum* K13-propeller polymorphisms. N Engl J Med.

[13] Imwong M, Hanchana S, Malleret B, Rénia L, Day NP, Dondorp A (2014). High-throughput ultrasensitive molecular techniques for quantifying low-density malaria parasitemias. J Clin Microbiol.

[14] Adams M, Joshi SN, Mbambo G, Mu AZ, Roemmich SM, Shrestha B (2015). An ultrasensitive reverse transcription polymerase chain reaction assay to detect asymptomatic low-density *Plasmodium falciparum* and *Plasmodium vivax* infections in small volume blood samples. Malar J..

[15] Imwong M, Nguyen TN, Tripura R, Peto TJ, Lee SJ, Lwin KM (2015). The epidemiology of subclinical malaria infections in South-East Asia: findings from cross-sectional surveys in Thailand-Myanmar border areas, Cambodia, and Vietnam. Malar J..

[16] Imwong M, Stepniewska K, Tripura R, Peto TJ, Lwin KM, Vihokhern B (2016). Numerical distributions of parasite densities during asymptomatic malaria. J Infect Dis.

[17] Zainabadi K, Adams M, Han ZY, Hnin WL, Han KT, Ouattara A (2017). A novel method for extracting nucleic acids from dried blood spots for ultrasensitive detection of low-density *Plasmodium falciparum* and *Plasmodium vivax* infections. Malar J..

[18] Schwartz A, Baidjoe A, Rosenthal PJ, Dorsey G, Bousema T, Greenhouse B (2015). The effect of storage and extraction methods on amplification of *Plasmodium falciparum* DNA from dried blood spots. Am J Trop Med Hyg.

[19] Oyola SO, Ariani CV, Hamilton WL, Kekre M, Amenga-Etego LN, Ghansah A (2016). Whole genome sequencing of *Plasmodium falciparum* from dried blood spots using selective whole genome amplification. Malar J..

