# Supplementary material for: An improved nucleic acid extraction method from dried blood spots for amplification of Plasmodium falciparum kelch13 for detection of artemisinin resistance
Source: Malar J. 2019 Jun 11;18:192. doi: 10.1186/s12936-019-2817-8 (PMC6558694; doi:10.1186/s12936-019-2817-8)
Supplement: Supplementary file 1 — Additional file 1. The final k13 dried blood spot (DBS) extraction protocol. [file 12936_2019_2817_MOESM1_ESM.docx]

**Additional file 1 The final** *k13* **dried blood spot (DBS) extraction protocol**

|  | **Home-made***  **Buffers** | **Commercial**  **Buffer Substitutes** |
| --- | --- | --- |
| **Lysis:** | 3M Guanidine thiocyanate  16.7% Isopropanol  2% Triton X100  10mM EDTA  5mM Trizma HCl pH 7.4  0.1% 6N HCl  0.5% 2-mercaptoethanol  pH 6.0-6.5 | Qiagen RLT-plus  16.7% Isopropanol  0.5% 2-mercaptoethanol |
| **Wash 1:** | Same as lysis but no  2-mercaptoethanol | Same as lysis but no  2-mercaptoethanol |
| **Wash 2:** | 25% Ethanol  25% Isopropanol  100mM Sodium Chloride  10mM Trizma HCl pH 7.4 | 70% ethanol, 30% PBS |

NOTE: Lysis and Wash 1 contain guanidine thiocyanate which is **TOXIC** and **INCOMPATIBLE** with bleach. Lysis contains 2-mercaptoethanol which is **TOXIC** and should be used in a fume hood.

***** Instructions on how to make large batches of these buffers (and proper contamination control measures), as well as sources of all reagents/materials have been previously published [17].

1. Cut a 50µl DBS into small enough pieces to fit into a 96 well deep well plate
   1. Take proper decontamination precautions to prevent cross-contamination from cutting: https://www.youtube.com/watch?v=K129KmLh_Ok
2. Add 900µl of lysis buffer using a multichannel pipette
3. Seal plate forcefully and centrifuge briefly to collect samples to the bottom
4. Place in shaking incubator for 2 hours (60-65 °C, 250RPM)
5. While waiting, place Nunc 96^1,2^ well DNA plate on top of a 2ml 96 well deep well plate
6. After incubation is finished, centrifuge briefly to collect samples to the bottom
7. Transfer 750µl of the lysate to Nunc DNA plate
   1. Use of plate covers will help prevent contamination from pipetting at this step
8. Spin 3,700RPM for 1 minute
9. Pipette 500µl of Wash 1
10. Spin 3,700RPM for 1 minute
11. Pipette 500µl of Wash 2
12. Spin 3,700RPM for 2 minute
13. Place DNA plate in 60-65 °C for 10 minutes to dry
14. Place DNA plate on top of 0.5ml 96 well plate
15. Pipette 50µl of TE buffer pH 8.0
16. Spin 3,700RPM for 2 minute

^1^ Omega EZ 96-well DNA plates are also compatible, though display slightly reduced sensitivity for *k13* amplification at parasitaemias below 5,000 parasites/mL.

^2^ If performing only a few extractions, individual DNA spin columns are also available from Omega Biotek (VWR 95043-210).
