# Supplementary material for: An improved nucleic acid extraction method from dried blood spots for amplification of Plasmodium falciparum kelch13 for detection of artemisinin resistance
Source: Malar J. 2019 Jun 11;18:192. doi: 10.1186/s12936-019-2817-8 (PMC6558694; doi:10.1186/s12936-019-2817-8)
Supplement: Supplementary file 2 — Additional file 2. Longer incubation times improve recovery of human, but not parasite DNA. [file 12936_2019_2817_MOESM2_ESM.docx]

**Additional file 2 Longer incubation times improve recovery of human, but not parasite DNA**

|  | 3M GuSCN + 16.7% ISOH | | |
| --- | --- | --- | --- |
| Incubation time at 65°C: | 1 hour | 2 hour | 4 hour |
| Average Ct for Pf 18S rDNA  (± SD) | 31.8  (±.4) | 31.5  (±.2) | 32.2  (±.2) |
| Average Ct for human actin  (± SD) | 27.2  (±.3) | 26.5  (±.2) | 26.5  (±.1) |

Experiments done with samples at parasite density of 2,000 parasites/mL;

Pf, *Plasmodium falciparum;* GuSCN, guanidine thiocyanate; ISOH, isopropanol; SD, standard deviation; Ct, cycle threshold
