# Supplementary material for: An improved nucleic acid extraction method from dried blood spots for amplification of Plasmodium falciparum kelch13 for detection of artemisinin resistance
Source: Malar J. 2019 Jun 11;18:192. doi: 10.1186/s12936-019-2817-8 (PMC6558694; doi:10.1186/s12936-019-2817-8)
Supplement: Supplementary file 3 — Additional file 3. Commercially available Qiagen RLT-plus buffer with 16.7% isopropanol performs similarly as the home-made lysis buffer. [file 12936_2019_2817_MOESM3_ESM.docx]

**Additional file 3. Commercially available Qiagen RLT-plus buffer with 16.7% isopropanol performs similarly as the home-made lysis buffer.**

| Reverse-transcriptase: | No | | Yes | |
| --- | --- | --- | --- | --- |
| Lysis buffer: | 3M GuSCN +  16.7% ISOH | RLT-plus +  16.7% ISOH | 3M GuSCN +  16.7% ISOH | RLT-plus +  16.7% ISOH |
| Average Ct for Pf 18S rDNA  (± SD) | 32.1  (±.5) | 32.3  (±.5) | 26.0  (±.5) | 26.1  (±.4) |

Experiments done with samples at parasite density of 1,000 parasites/mL; Pf, *Plasmodium falciparum;* GuSCN, guanidine thiocyanate; ISOH, isopropanol; SD, standard deviation; Ct, cycle threshold
