# Supplementary figures and images for: An improved nucleic acid extraction method from dried blood spots for amplification of Plasmodium falciparum kelch13 for detection of artemisinin resistance
Source: Malar J. 2019 Jun 11;18:192. doi: 10.1186/s12936-019-2817-8 (PMC6558694; doi:10.1186/s12936-019-2817-8)

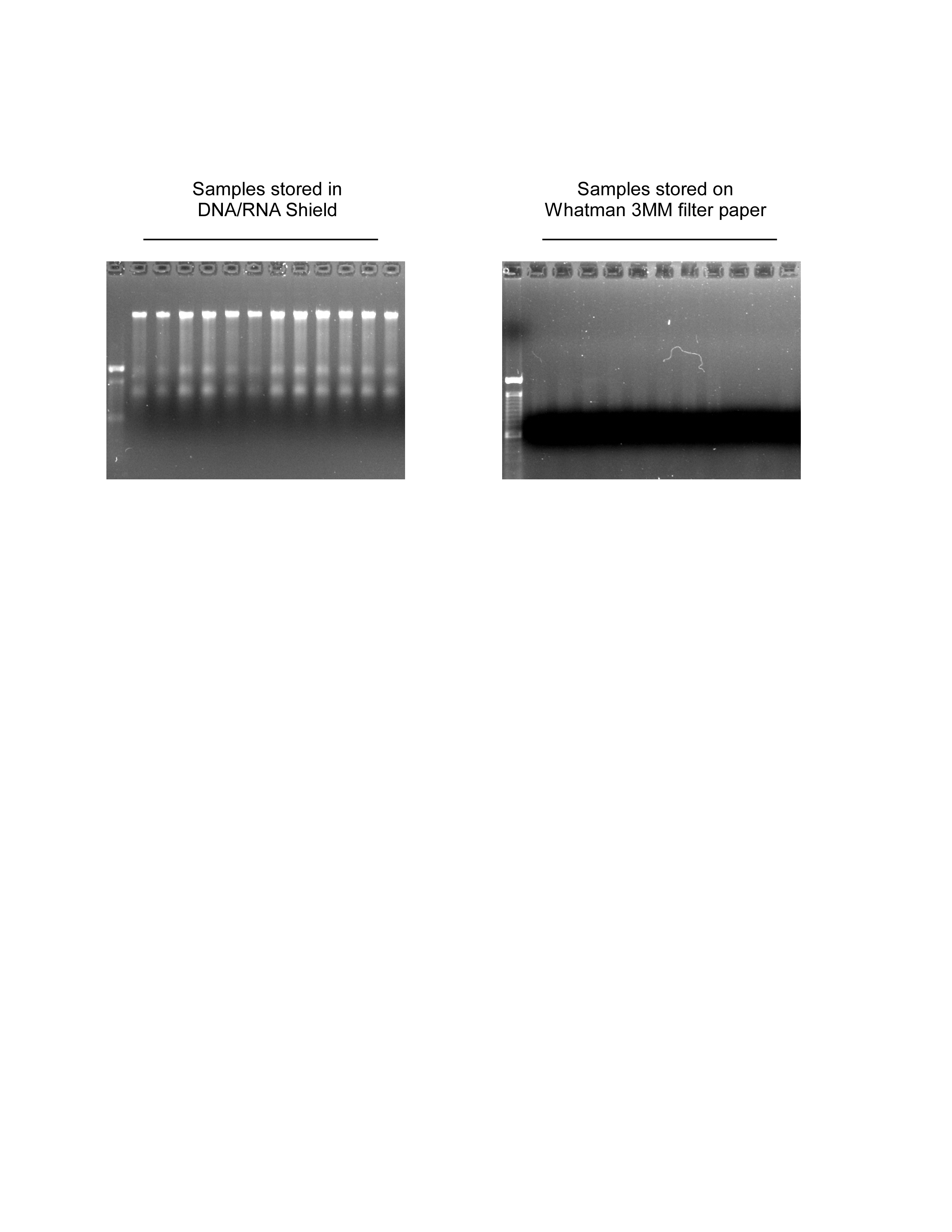

Supplement: Supplementary file 4 — Additional file 4. Evidence of nucleic acid degradation from samples stored on filter paper. [file 12936_2019_2817_MOESM4_ESM.tif]
